# Supplementary material for: Joint inference of exclusivity patterns and recurrent trajectories from tumor mutation trees
Source: Nat Commun. 2023 Jun 21;14:3676. doi: 10.1038/s41467-023-39400-w (PMC10284838; doi:10.1038/s41467-023-39400-w)
Supplement: Supplementary file 3 — Reporting Summary [file 41467_2023_39400_MOESM3_ESM.pdf]

nature portfolio

Corresponding author(s): Niko Beerenwinkel

Last updated by author(s): Apr 26, 2023

Reporting Summary

Nature Portfolio wishes to improve the reproducibility of the work that we publish. This form provides structure for consistency and transparency in reporting. For further information on Nature Portfolio policies, see our Editorial Policies and the Editorial Policy Checklist.

Statistics

For all statistical analyses, confirm that the following items are present in the figure legend, table legend, main text, or Methods section.

n/a	Confirmed	
☐	☒	The exact sample size (*n*) for each experimental group/condition, given as a discrete number and unit of measurement
☐	☒	A statement on whether measurements were taken from distinct samples or whether the same sample was measured repeatedly
☐	☒	The statistical test(s) used AND whether they are one- or two-sided<br>*Only common tests should be described solely by name; describe more complex techniques in the Methods section.*
☒	☐	A description of all covariates tested
☐	☒	A description of any assumptions or corrections, such as tests of normality and adjustment for multiple comparisons
☐	☒	A full description of the statistical parameters including central tendency (e.g. means) or other basic estimates (e.g. regression coefficient) AND variation (e.g. standard deviation) or associated estimates of uncertainty (e.g. confidence intervals)
☐	☒	For null hypothesis testing, the test statistic (e.g. F, t, r) with confidence intervals, effect sizes, degrees of freedom and P value noted<br>*Give P values as exact values whenever suitable.*
☒	☐	For Bayesian analysis, information on the choice of priors and Markov chain Monte Carlo settings
☒	☐	For hierarchical and complex designs, identification of the appropriate level for tests and full reporting of outcomes
☐	☒	Estimates of effect sizes (e.g. Cohen's d, Pearson's r), indicating how they were calculated

Our web collection on statistics for biologists contains articles on many of the points above.

Software and code

Policy information about availability of computer code

Data collection	R (v4.1.2), evoverse.datasets (v0.1.0), Rcpp (v1.0.10), RcppArmadillo (v0.11.4.4.0), dplyr (v1.1.0)
Data analysis	R (v4.1.2), TreeMHN (v1.0.0), Rcpp (v1.0.10), Matrix (v1.5-3), gtools (v3.9.4), DiagrammeR (v1.0.9), ggplot2 (v3.4.1), dplyr (v1.1.0), ggm (v2.5), reshape2 (v1.4.4), gridExtra (v2.3), ggpubr (v0.6.0), Rgraphviz (v2.40.0), revolver (v1.0.0), parallel (v4.2.1), devtools (v2.4.5), profmem (v0.6.0)

For manuscripts utilizing custom algorithms or software that are central to the research but not yet described in published literature, software must be made available to editors and reviewers. We strongly encourage code deposition in a community repository (e.g. GitHub). See the Nature Portfolio guidelines for submitting code & software for further information.

Data

Policy information about availability of data

All manuscripts must include a data availability statement. This statement should provide the following information, where applicable:
- Accession codes, unique identifiers, or web links for publicly available datasets
- A description of any restrictions on data availability
- For clinical datasets or third party data, please ensure that the statement adheres to our policy

The original sequencing data for the AML dataset [21] are available at NCBI BioProject ID PRJNA648656 [https://www.ncbi.nlm.nih.gov/Traces/study/?acc=PRJNA648656&o=acc_s%3Aa], and the associated mutation trees are provided with this paper. The original sequencing data for the NSCLC dataset [15] are available at the European Genome–Phenome Archive under accession code EGAS00001002247 [https://ega-archive.org/studies/EGAS00001002247], and the associated mutation trees are obtained from the R package evoverse.datasets v0.1.0 [https://github.com/caravagn/evoverse.datasets]. The original somatic

mutational and clinical data for the breast cancer dataset [32] are available at the cBioPortal for Cancer Genomics with study ID breast_msk_2018 [http://www.cbioportal.org/study?id=breast_msk_2018], and the associated mutation trees [9] are obtained from https://github.com/elkebir-group/RECAP/tree/master/data/breast_Razavi. All simulation data, processed mutation trees, and other relevant data are available as Source Data files at Zenodo [https://doi.org/10.5281/zenodo.7817793]. Source data are provided with this paper.

Human research participants

Policy information about studies involving human research participants and Sex and Gender in Research.

Reporting on sex and gender	This is not relevant, because our work uses existing datasets and does not directly involve human research participants.
Population characteristics	This is not relevant, because our work uses existing datasets and does not directly involve human research participants.
Recruitment	This is not relevant, because our work uses existing datasets and does not directly involve human research participants.
Ethics oversight	This is not relevant, because our work uses existing datasets and does not directly involve human research participants.

Note that full information on the approval of the study protocol must also be provided in the manuscript.

Field-specific reporting

Please select the one below that is the best fit for your research. If you are not sure, read the appropriate sections before making your selection.

[x] Life sciences [ ] Behavioural & social sciences [ ] Ecological, evolutionary & environmental sciences

For a reference copy of the document with all sections, see nature.com/documents/nr-reporting-summary-flat.pdf

Life sciences study design

All studies must disclose on these points even when the disclosure is negative.

Sample size	For synthetic datasets, different sample sizes (200, 300, 500) were chosen to show the associated impact on parameter estimation accuracy. The sample sizes of the real datasets were determined in the original studies (Morita, K. et al., 2020; Jamal-Hanjani, M. et al., 2017; Razavi, P. et al., 2018).
Data exclusions	For the advanced breast cancer dataset (Razavi, P. et al., 2018), we excluded primary tumor samples with treatments to avoid confounding effects. More details can be found in supplementary information.
Replication	In simulations, we perform 100 random independent replications for each configuration of the experiments. In real data analyses, we perform inference by randomly subsampling the mutation trees 1000 times in order to prevent overfitting to the data and obtain stable results.
Randomization	This is not relevant, because our work does not have an experimental design that involves allocations of samples into groups.
Blinding	This is not relevant, because our work does not have an experimental design that involves allocations of samples into groups.

Reporting for specific materials, systems and methods

We require information from authors about some types of materials, experimental systems and methods used in many studies. Here, indicate whether each material, system or method listed is relevant to your study. If you are not sure if a list item applies to your research, read the appropriate section before selecting a response.

Materials & experimental systems

n/a	Involved in the study	
[x]	[ ]	Antibodies
[x]	[ ]	Eukaryotic cell lines
[x]	[ ]	Palaeontology and archaeology
[x]	[ ]	Animals and other organisms
[x]	[ ]	Clinical data
[x]	[ ]	Dual use research of concern

Methods

n/a	Involved in the study	
[x]	[ ]	ChIP-seq
[x]	[ ]	Flow cytometry
[x]	[ ]	MRI-based neuroimaging
